# Transcranial sonography changes in heterozygotic carriers of the *ATP7B* gene

**DOI:** 10.1007/s10072-020-04378-6

**Published:** 2020-04-09

**Authors:** Marta Skowronska, Tomasz Litwin, Iwona Kurkowska-Jastrzębska, Anna Członkowska

**Affiliations:** 1 2nd Department of Neurology, Institute of Psychiatry and Neurology, 02-957 Warsaw, Poland; 2 Department of Experimental and Clinical Pharmacology, Medical University of Warsaw, Warsaw, Poland

**Keywords:** Transcranial sonography, Wilson’s disease, Heterozygotic carriers, Basal ganglia

## Abstract

**Purpose:**

Wilson’s disease (WD) is an autosomal recessive disorder of *ATP7B* gene leading to impaired copper metabolism. Brain imaging, such as magnetic resonance (MR) and transcranial sonography (TCS) in WD patients, shows changes mostly in the basal ganglia. Heterozygotic carriers of one faulty *ATP7B* gene should not exhibit symptoms of WD, but one in three heterozygotes has copper metabolism abnormalities. This study examined heterozygote *ATP7B* mutation carriers using TCS to assess any basal ganglia changes compared with healthy controls.

**Methods:**

Heterozygote carriers and healthy volunteers underwent the same standard MR and TCS imaging protocols. Heterozygotes were followed for 5 years and monitored for the development of neurological symptoms.

**Results:**

The study assessed 34 heterozygotes (21 women), with mean age of 43 years (range of 18 to 74 years) and 18 healthy controls (13 women), with mean age of 47 years (range of 20 to 73 years). Bilateral lenticular nucleus (LN) hyperechogenicity was found in 25 heterozygotes, but none of the controls (*p* < 0.001). Bilateral substantia nigra (SN) hyperechogenicity was found in 8 heterozygotes and one control; another 3 heterozygotes had unilateral SN hyperechogenicity (*p* = 0.039 for the right; *p* = 0.176 for the left). Heterozygotes had larger SN area on both sides compared with controls (*p* = 0.005 right; *p* = 0.008 left).

**Conclusions:**

SN and LN hyperechogenicity were more frequent in heterozygotes than in controls, probably due to copper accumulation, but it remains unknown if this predisposes to brain neurodegeneration.

## Introduction

Wilson’s disease (WD) is an autosomal recessive inherited disorder of hepatic copper metabolism that is caused by malfunction of a putative copper-transporting P-type ATPase, ATP7B. Cellular damage occurs due to copper deposition in affected tissues, principally the liver and the brain [1, 2]. Clinical manifestations of WD include hepatic, neurological with predominantly extrapyramidal symptoms and psychiatric symptoms. Brain magnetic resonance (MR) imaging (MRI) in most WD patients with the neuropsychiatric form, and some with hepatic and presymptomatic forms, show hyperintense lesions in the putamen, pons, midbrain, and thalamus together with hypointense changes in the basal ganglia, mostly in the globus pallidus (GP), red nucleus, and substantia nigra (SN) on T2-weighted images [3–7]. Transcranial sonography (TCS), a simple and safe technique applied for the diagnosis of several neurodegenerative diseases, also shows changes in the deep brain nuclei of WD patients [8–10].

The number of WD heterozygotic carriers—having only one faulty copy of the *ATP7B* gene—is high, at around 1 in 90 individuals or 1–2% of the general population [11, 12]. However, the latest molecular data from the UK estimate that the number of heterozygotic carriers could potentially be up to 2.5% of the general population [13]. Heterozygotic carriers of the *ATP7B* gene should not exhibit symptoms of the disease, but copper metabolism abnormalities have been noted, in a range from 20% of cases [14], with up to 28.6% having low ceruloplasmin, and 35% exhibiting low serum copper levels [15, 16]. Conventional MR techniques do not show any structural changes in the basal ganglia of heterozygotes; however, magnetic resonance spectroscopy (MRS) revealed changes in the GP and thalamus suggestive of neuronal energy impairment, gliosis and copper accumulation [17].

The aim of this study was to examine heterozygote carriers of the *ATP7B* gene with TCS to assess any basal ganglia changes and to compare with healthy controls.

## Methods

### Subjects and assessments

Heterozygote carriers of the *ATP7B* gene were mostly recruited from the relatives of WD patients admitted for family screening for presymptomatic WD. All included heterozygotes underwent the same assessments including medical history, neurological examination, routine laboratory screening (e.g., elevated liver enzymes, morphology abnormalities), copper metabolism screening (24-h urine copper excretion, serum ceruloplasmin, and copper levels), DNA analysis, and brain imaging using standard MRI and TCS protocols. The methods used for copper metabolism and DNA analysis were as described previously [16]. Heterozygote carriers were followed up after 5 years for neurological symptoms or disability complaints.

The control group consisted of healthy volunteers who had no abnormalities in routine laboratory tests and no neurological abnormalities. They underwent the same standard MR and TCS imaging examinations. Genetic sampling and copper metabolism were not conducted in this group.

All procedures performed in studies involving human participants were in accordance with the ethical standards of the institutional and/or national research committee and with the 1964 Helsinki declaration and its later amendments or comparable ethical standards.

### Image acquisition and processing

In both groups, MRI was performed using a 1.5-T scanner (Philips Achieva, Eindhoven,Netherlands). T1-weighted (TR = 596 ms, TE = 15 ms) and T2-weighted (TR = 6783 ms, TE = 140 ms) images were acquired in axial planes with 5-mm slice thickness. Gradient echo T2* images were obtained as a single-echo sequence (TR = 693 ms, TE = 23 ms, flip angle = 20°). The imaging results were assessed by a neuroradiologist. Changes in the basal ganglia, mostly in the GP, putamen, thalamus, and SN, were recorded.

**Fig. 1:**
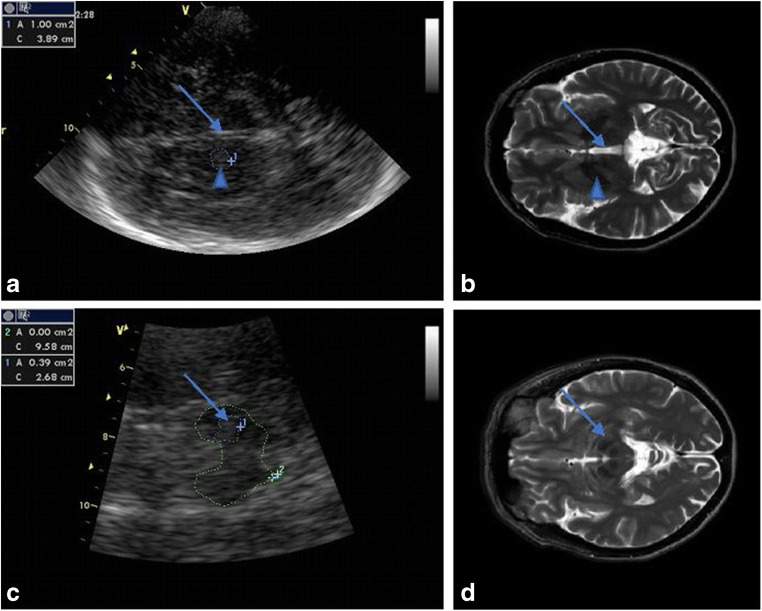
Midbrain structures in transcranial sonography (TCS) with corresponding magnetic resonance imaging (MRI) T2-weighted images. **a** TCS with the hyperechogenic part of the LN measured (arrowhead). Arrow showing the 3rd ventricle, **b** Corresponding MR results. **c** TCS with substantia nigra measured (arrow). **d**. Corresponding MR results

In both groups, TCS was performed through the preauricular acoustic bone window, using a 2.5-MHz phased-array transducer (Vivid 7; GE, Wisconsin USA) by a sonographer (MS) who was blinded to the MRI results. The sonographer was also blinded to the genetic results at the time of ultrasound examination. The chosen ultrasound parameters were penetration depth of 14–16 cm and a dynamic range of 50 dB. Image contrast and brightness were adjusted to obtain the best image. SN echogenic size measurements were performed automatically on axial TCS scans after manually encircling the outer circumference of the SN echogenic area. For the ultrasound system used, SN echogenic areas of ≥ 0.25 cm^2^ were considered hyperechogenic, those ≤ 0.23 cm^2^ were considered normal, and intermediate-sized areas were considered moderately hyperechogenic. The GP and putamen were assessed as one structure, the LN, as in the TCS protocols. The LN and thalamus were visualized in the third ventricular plane. The echogenicity of the LN and thalamus were classified as hyperechogenic if echogenicity was more intense than the surrounding white matter. The area of hyperechogenicity was measured by manually encircling the outer circumference of the hyperechogenic area. The width of the third ventricle was measured on the axial scanning plane, with width < 0.7 cm considered normal (Fig. 1). Since the study design involved recording two measurements for both sides, we present the result for both sides.

### Statistical analysis

Statistical analysis was performed using SAS version 13.2. A *p* value of < 0.05 was considered to indicate statistical significance. Wilcoxon Test and Fisher’s Exact Test were used to compare heterozygotes and controls. Logistic regression models were used to investigate the relationship between copper metabolism and LN and SN hyperechogenicity.

## Results

The study group consisted of 34 heterozygote carriers of the *ATP7B* gene; 21 were women (61.7%), with a mean age of 43 years (range from 18 to 74 years). The mutations found in the study group were as follows: p.H1069Q (c. 3207C > A) in 28 cases, p.IVS162A > G (c.3557-2A > G) in 3 cases, p.Q1351X (c.4051C > T) in 2 cases, and p.A1135fs (3402 delC) in 1 case.

Ceruloplasmin levels below the laboratory range were found in 11 cases, and mean level was 29.16 ± 9.02 mg/dL. Low copper plasma levels were observed only in 5 cases, and mean serum copper was 91.91 ± 30.10 μg/dL. In all cases, 24-h urine copper excretion levels were normal, with mean urinary excretion 23.72 ± 28.82 μg/24 h. Routine laboratory results did not reveal any abnormalities. MR changes were found in T2* sequences in 2 cases: in the first case, there was bilateral hypointensity in the GP and in the second case, there was bilateral hypointensity in the SN. Both had p.H1069Q mutations.

The control group consisted of 18 healthy subjects; 13 were women (72.2%), with a mean age was 47 years (range from 20 to 73 years). There were no differences in sex and age distribution between heterozygote and control groups (*p* = 0.45 for sex and *p* = 0.38 for age). All controls had normal MRI results.

**Fig. 2:**
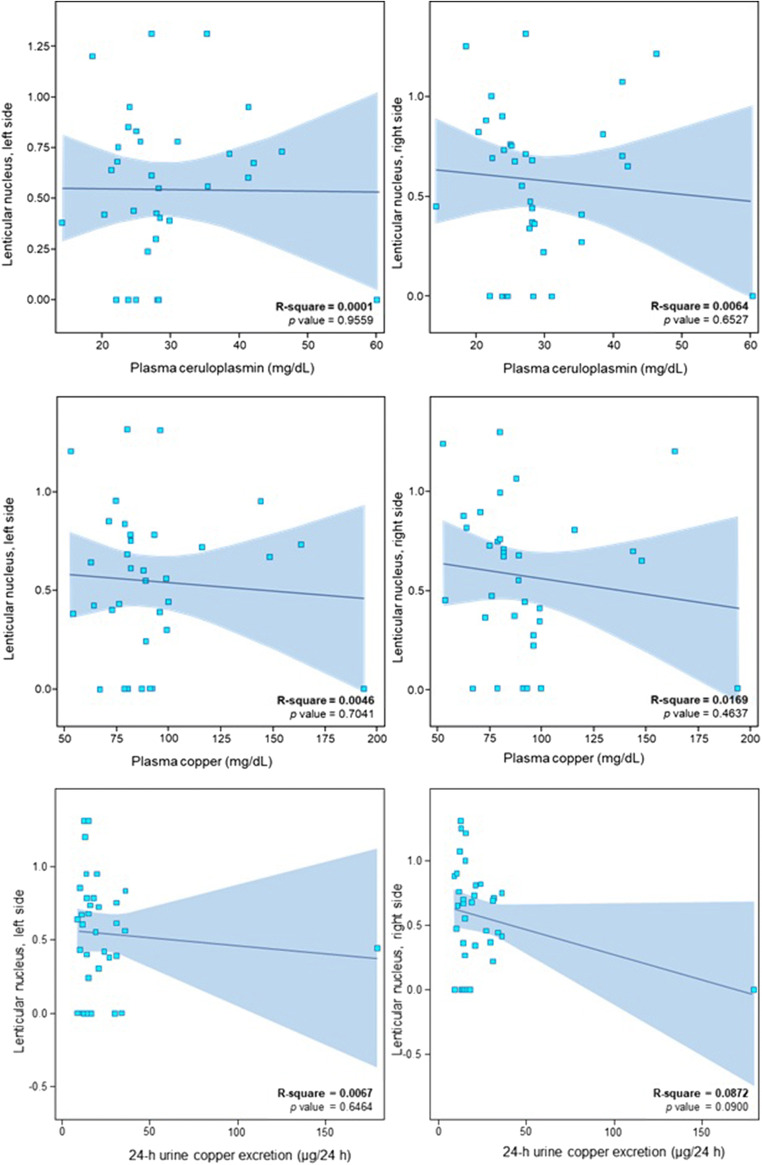
Regression model for lenticular nucleus (LN) echogenicity and ceruloplasmin level, serum copper level and 24-h urine copper excretion. **a** LN hyperechogenicity and ceruloplasmin level. **b** LN hyperechogenicity and serum copper level. **c** LN hyperechogenicity and 24-h urine copper excretion

**Fig. 3:**
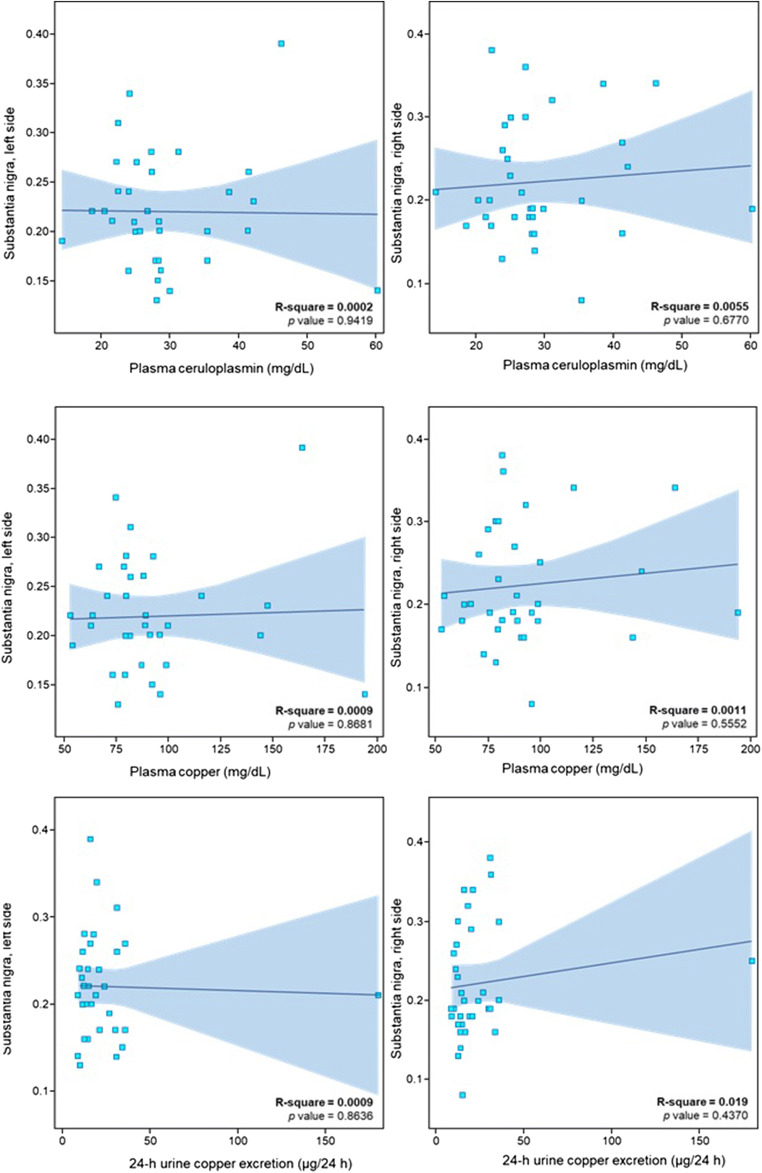
Regression model for substantia nigra (SN) echogenicity and copper metabolism. **a** SN hyperechogenicity and ceruloplasmin level. **b** SN hyperechogenicity and serum copper level. **c** SN hyperechogenicity and 24-h urine copper excretion

TCS results for the heterozygote carriers and controls are shown in Table 1. Bilateral LN hyperechogenicity was found in 25 heterozygote carriers, but none of the control cases (*p* < 0.001). Unilateral changes were present in 5 control cases. Mean LN echogenicity area was larger for heterozygote carriers compared with controls (*p* = 0.001 for right and *p* = 0.002 for left). Depending on the type of mutation, mean LN echogenicity area was as follows: for p.H1069Q, 0.52 cm^2^ and 0.51cm^2^; for p.IVS162A > G, 1.05 cm^2^ and 1.12cm^2^; for p.Q1351X, 0.22 cm^2^ and 0 cm^2^; and for the only patient with p.A1135fs, 1.0 cm^2^ and 0.68 cm^2^, respectively, for the right and left side. Bilateral SN hyperechogenicity was found in 8 heterozygote carriers and one control, while unilateral SN hyperechogenicity was present in 3 heterozygote carriers and no controls (*p* = 0.039 for the right; *p* = 0.176 for the left). Heterozygote carriers had larger SN area on both sides compared with controls (*p* = 0.005 for the right; *p* = 0.008 for the left). Thalamic hyperechogenicity was found in 3 heterozygote carriers and no controls, but the difference between the groups was not significant (*p* = 0.19). The width of the third ventricle was within the normal range in all heterozygote carriers (< 0.7 cm) and in 15 of the control cases. Controls had wider third ventricles than heterozygote carriers (*p* = 0.016). There was no correlation between TCS LN or SN hyperechogenicity and ceruloplasmin level, copper plasma level, and urinary copper excretion (Figs. 2 and 3).

**Table 1:** Transcranial sonography results from the substantia nigra (SN) and lenticular nucleus (LN) (right [R] and left [L] sides) of heterozygotic *ATP7B* carriers and healthy controls

	Heterozygote carriers	Controls	*p*
Midbrain structure (area in cm^2^, mean ± SD)			
SN R	0.222 ± 0.07	0.167 ± 0.05	0.005
LN R	0.572 ± 0.378	0.041 ± 0.174	<0.001
SN L	0.220 ± 0.058	0.174 ± 0.049	0.008
LN L	0.543 ± 0.377	0.16 ± 0.316	0.002
Third ventricle width (cm, mean ± SD)	0.305 ± 0.134	0.434 ± 0.197	0.016
SN hyperechogenicity (area in cm^2^, no of cases)			
SN R < 0.23	21	17	0.039*
SN R 0.23–0.25	3	0	
SN R > 0.25	10	1	
SN L < 0.23	21	15	0.176*
SN L 0.23–0.25	4	2	
SN L > 0.25	9	1	

*p value for the whole group

*SD*, standard deviation

Two heterozygotes were lost to follow up over the 5 years of the study. From the 32 followed heterozygote cases, 2 had neurological symptoms or complaints. One female heterozygote carrier with SN and LN hyperechogenicity on both sides on TCS developed amyotrophic lateral sclerosis (ALS). A second female heterozygote carrier complained of memory problems—this case had no SN hyperechogenicity but bilateral LN hyperechogenicity on TCS. None of the followed cases developed extrapyramidal symptoms.

## Discussion

TCS has gathered great attention in different neurodegenerative disorders. It has become an additional diagnostic tool for idiopathic Parkinson’s disease (PD) as hyperechogenicity of the SN is observed in about 90% of patients in the very early clinical stages of PD [18]. TCS seems to be a more sensitive imaging tool than MR, showing basal ganglia changes even in patients with normal MR results [8].

LN hyperechogenicity is the most common TCS abnormality in WD, found in all patients with the neurological form of WD studied previously [8, 9]. In the hepatic form of WD, LN hyperechogenicity is less common and present in about quarter of patients [9], but it has been detected even in asymptomatic patients [8]. SN hyperechogenicity is also found in WD patients but is less common than LN hyperechogenicity. SN hyperechogenicity is found in about 30–50% of patients, most often in those with neurological symptoms [8, 9]. LN and SN hyperechogenicity have been shown to correlate with disease severity in patients with WD [8, 9]. Less common changes are red nucleus hyperechogenicity, thalamic hyperechogenicity, and dilatation of the third ventricle.

In the current study, we found hyperechogenicity of LN and SN in heterozygote *ATP7B* carriers, who had normal structural MR. In our study, almost three-quarters of heterozygotes carriers had bilateral LN hyperechogenicity, while bilateral LN hyperechogenicity was not found in the control group. There might be LN echogenicity area differences, depending on type of mutation—with the highest for p.IVS162A > G and lowest p.Q1351X. Since the groups were very small this is not necessarily true and no statistical analysis was performed.

Heterozygotes also had marked bilateral SN hyperechogenicity in about one-quarter of cases. Hyperechogenicity of SN is the imaging phenomenon typical for the PD, present in more than 90% of patients, but only in approximately 10% persons without PD [19]. Hyperechogenicity of the SN is a risk marker for PD, increasing the risk 20 times over a 5-year follow-up period [20]. The number of heterozygotes with SN hyperechogenicity in our study population was higher than average, but over 5 years’ follow-up, none of them developed extrapyramidal symptoms. One patient, with bilateral marked SN hyperechogenicity developed ALS. SN hyperechogenicity is found in about 70% ALS patients [21]. The functional role of SN pathology in ALS is still unclear. It remains uncertain at this point whether the degenerative process of the SN in ALS is comparable with PD [21]. Our finding suggests that neurodegenerative process involving SN starts before clinical symptoms develop.

Thalamic TCS hyperechogenicity was found only in 3 heterozygote carriers, with no differences between heterozygotes and controls. Thalamic hyperechogenicity has been previously described in WD patients [8], but there are also studies that do not support this observation [9].

One of the markers of neurodegeneration and brain atrophy is the width of the third ventricle, with many WD patients exhibiting dilatation of the third ventricle. Neurological disease severity, assessed with the United Wilson Disease Rating Scale, has been shown to correlate with the widths of the third ventricle on TCS [8, 9]. In our study, none of the heterozygote carriers and 3 controls had moderate dilatation of the third ventricle, and the average third ventricle width was higher in the control group. This suggests that none of the heterozygote carriers had brain atrophy as observed in WD patients.

TCS findings in healthy heterozygote carriers of the *ATP7B* gene raise questions regarding the nature of hyperechogenicity of deep brain nuclei, with the exact answer still unknown. TCS hyperechogenicity was first described in a small group of PD patients, showing changes in SN, which was later attributed to iron accumulation [22]. TCS studies in patients with brain metal accumulation support that observation. TCS studies of patients with neurodegeneration with brain iron accumulation (NBIA), mostly pantothenate kinase-associated neurodegeneration (PKAN) and membrane protein-associated neurodegeneration (MPAN) patients [23, 24], WD patients [8, 9, 25], and manganese-induced parkinsonism [26, 27] patients, have all shown TCS changes, probably due to iron, copper, and manganese accumulation. In ALS and idiopathic dystonia, iron or copper accumulation, respectively, have been described in deep brain nuclei [21, 28]. Hyperechogenicity of the LN is also found in more than 75% of patients with primary cervical and upper-limb dystonia, and in 31% of those with facial dystonia [29, 30]. Postmortem examination of patients with primary dystonia found significantly increased copper and manganese levels in the GP and putamen in comparison with control brain samples [28]. Postmortem TCS studies of WD patients showed a correlation of putaminal copper concentration with LN area [25].

We speculate that LN and SN hyperechogenicity in heterozygotes are also due to copper accumulation, but we failed to find a correlation with copper metabolism and TCS findings. Thus, it is possible that hyperechogenicity is influenced by not only metal ions themselves but also different ion compounds and binding partners. Experimental studies in an animal model of PD show that SN hyperechogenicity is caused by structural changes, such as gliosis, rather than by increased iron concentration [31].

To conclude, this is the first study, to our knowledge, of heterozygote carriers of an *ATP7B* mutation showing TCS basal ganglia changes in cases with normal MR results and neurological examination. We found that SN and LN hyperechogenicity were more common in heterozygote carriers than in controls and this is probably due to copper accumulation. It remains unknown if the SN and LN hyperechogenicity observed can predispose heterozygotes to brain neurodegeneration. In general population hyperechogenicity of the SN is a risk marker for PD, but in heterozygote, *ATP7B* carriers is not a risk factor for developing PD/parkinsonian syndrome over 5 years follow-up.
